# Effect of a Community-Based Nursing Intervention on Mortality in Chronically Ill Older Adults: A Randomized Controlled Trial

**DOI:** 10.1371/journal.pmed.1001265

**Published:** 2012-07-17

**Authors:** Kenneth D. Coburn, Sherry Marcantonio, Robert Lazansky, Maryellen Keller, Nancy Davis

**Affiliations:** Health Quality Partners, Doylestown, Pennsylvania, United States of America; University of Cambridge, United Kingdom

## Abstract

**Background:**

Improving the health of chronically ill older adults is a major challenge facing modern health care systems. A community-based nursing intervention developed by Health Quality Partners (HQP) was one of 15 different models of care coordination tested in randomized controlled trials within the Medicare Coordinated Care Demonstration (MCCD), a national US study. Evaluation of the HQP program began in 2002. The study reported here was designed to evaluate the survival impact of the HQP program versus usual care up to five years post-enrollment.

**Methods and Findings:**

HQP enrolled 1,736 adults aged 65 and over, with one or more eligible chronic conditions (coronary artery disease, heart failure, diabetes, asthma, hypertension, or hyperlipidemia) during the first six years of the study. The intervention group (*n* = 873) was offered a comprehensive, integrated, and tightly managed system of care coordination, disease management, and preventive services provided by community-based nurse care managers working collaboratively with primary care providers. The control group (n = 863) received usual care. Overall, a 25% lower relative risk of death (hazard ratio [HR] 0.75 [95% CI 0.57–1.00], *p* = 0.047) was observed among intervention participants with 86 (9.9%) deaths in the intervention group and 111 (12.9%) deaths in the control group during a mean follow-up of 4.2 years. When covariates for sex, age group, primary diagnosis, perceived health, number of medications taken, hospital stays in the past 6 months, and tobacco use were included, the adjusted HR was 0.73 (95% CI 0.55–0.98, *p* = 0.033). Subgroup analyses did not demonstrate statistically significant interaction effects for any subgroup. No suspected program-related adverse events were identified.

**Conclusions:**

The HQP model of community-based nurse care management appeared to reduce all-cause mortality in chronically ill older adults. Limitations of the study are that few low-income and non-white individuals were enrolled and implementation was in a single geographic region of the US. Additional research to confirm these findings and determine the model's scalability and generalizability is warranted.

**Trial Registration:**

ClinicalTrials.gov NCT01071967

*Please see later in the article for the Editors' Summary*

## Abstract 2

Kenneth Coburn and colleagues report findings from a randomized trial evaluating the effects of a complex nursing intervention on mortality risk among older individuals diagnosed with chronic health conditions.

## Editors' Summary

**Background:**

In almost every country in the world, the proportion of people aged over 60 years is growing faster than any other age group because of increased life expectancy. This demographic change has several implications for public health, especially as older age is a risk factor for many chronic diseases—diseases of long duration and generally slow progression. Chronic diseases, such as heart disease, stroke, cancer, chronic respiratory diseases, and diabetes, are by far the leading cause of death in the world, representing almost two-thirds of all deaths. Therefore in most countries, the challenge of managing increasingly ageing populations who have chronic illnesses demands an urgent response and countries such as the United States are actively researching possible solutions.

**Why Was This Study Done?:**

Some studies suggest that innovations in chronic disease management that are led by nurses may help address the epidemic of chronic diseases by increasing the quality and reducing the cost of care. However, to date, reports of the evaluation of such interventions lack rigor and do not provide evidence of improved long-term health outcomes or reduced health care costs. So in this study, the researchers used the gold standard of research, a randomized controlled trial, to examine the impact of a community-based nurse care management model for older adults with chronic illnesses in the United States as part of a series of studies supported by the Centers for Medicare and Medicaid Services.

**What Did the Researchers Do and Find?:**

The researchers recruited eligible patients aged 65 years and over with heart failure, coronary heart disease, asthma, diabetes, hypertension, and/or hyperlipidemia who received traditional Medicare—a fee for service insurance scheme in which beneficiaries can choose to receive their care from any Medicare provider—from participating primary care practices in Pennsylvania. The researchers then categorized patients according to their risk on the basis of several factors including the number of chronic diseases each individual had before randomizing patients to receive usual care or the nurse-led intervention. The intervention included an individualized plan comprising education, symptom monitoring, medication, counseling for adherence, help identifying, arranging, and monitoring community health and social service referrals in addition to group interventions such as weight loss maintenance and exercise classes. The researchers checked whether any participating patients had died by using the online Social Security Death Master File. Then the researchers used a statistical model to calculate the risk of death in both groups.

Of the 1,736 patients the researchers recruited into the trial, 873 were randomized to receive the intervention and 863 were in the control group (usual care). The researchers found that 86 (9.9%) participants in the intervention group and 111 (12.9%) participants in the control group died during the study period, representing a 25% lower relative risk of death among the intervention group. However, when the researchers considered other factors, such as sex, age group, primary diagnosis, perceived health, number of medications taken, hospital stays in the past 6 months, and tobacco use in their statistical model, this risk was slightly altered—0.73 risk of death in the intervention group.

**What Do These Findings Mean?:**

These findings suggest that that community-based nurse care management is associated with a reduction in all-cause mortality among older adults with chronic illnesses who are beneficiaries of the fee for service Medicare scheme in the United States. These findings also support the important role of nurses in improving health outcomes in this group of patients and show the feasibility of implementing this program in collaboration with primary care practices. Future research is needed to test the adaptability, scalability, and generalizability of this model of care.

**Additional Information:**

Please access these Web sites via the online version of this summary at http://dx.doi.org/10.1371/journal.pmed.1001265.

- This study is further discussed in a *PLoS Medicine* Perspective by Arlene Bierman
- Information about the Centers for Medicare and Medicaid Services is available
- The World Health Organization provides statistics on the prevalence of both chronic illness and ageing
- Heath Quality Partners provide information about the study

## Introduction

Chronically ill older adults have complex patterns of health care, frequent hospital readmissions, often receive poor or inconsistent quality of care, and account for the majority of health care expenditures in the United States [1]–[6]. Long appreciated as the dominant disease burden in upper-income countries, noncommunicable chronic disease is now recognized as a major global health problem [7],[8].

Several leading organizations and experts argue that care coordination emphasizing wellness, prevention, and chronic disease management is a promising means to increase the quality and perhaps reduce the costs of care for chronic illness in the elderly [9]–[11]. Broader testing and use of chronic disease management interventions in several countries have resulted in reports describing the challenges associated with such efforts, but very few have provided evidence of improved long-term health outcomes or reduced health care expenditures [12]–[22]. Positive reports that have been published have often come from non-experimental evaluations of clinic or practice-based interventions targeting a single chronic disease [21],[22]. Some researchers believe that innovations in nursing-led chronic disease management may help address chronic disease in areas of the world with less abundant health care resources, such as sub-Saharan Africa [23],[24].

Based on research published to date, there is uncertainty about whether nurse care management programs have the potential to improve the long-term health outcomes of chronically ill older adults. In this study we report outcomes of a longitudinal community-based nurse care management model on all-cause mortality, using a randomized controlled design.

Support for this research came from the Medicare Coordinated Care Demonstration (MCCD), a national study in the United States administered by the Centers for Medicare and Medicaid Services (CMS), which sponsored 15 unique longitudinal, prospective, randomized, controlled trials [25]. Since 2002, the MCCD has independently tested these different, competitively selected, care coordination programs in an attempt to identify specific models that lower health care costs and improve quality among US Medicare beneficiaries (fee for service coverage) with chronic conditions.

Descriptions of the varied programs that participated in the MCCD have been published elsewhere by Mathematica Policy Research, Inc. (MPR), the contracted evaluator for the demonstration [26],[27]. The programs selected to participate in the MCCD varied in terms of the number and types of chronic conditions they targeted. Six programs targeted a single condition, three enrolled patients using criteria other than specific diagnoses, and six targeted multiple conditions with or without additional targeting criteria. The most common primary diagnoses of patients enrolled across all programs were heart failure, coronary heart disease, and diabetes. The 15 organizations that implemented programs in the MCCD were also diverse and included four commercial disease management vendors, three hospitals, three academic medical centers, one integrated delivery system, one hospice program, one long-term care facility, one retirement community, and one health care quality research and development organization.

The interventions offered by the programs in the MCCD varied, though all programs used care coordinators, which were typically registered nurses (only one program used licensed practical nurses). Nearly all programs educated patients in order to improve medication adherence, diet, exercise, and self-care. Fourteen programs sought to coordinate care for patients through a variety of mechanisms. Ten programs had timely data on hospitalizations and emergency room visits to support interventions related to the transitions of care. Fourteen programs relied on patients to provide care coordinators with a list of medications they were taking. Four programs focused on increasing physicians' adherence to evidence-based or guideline-based care. In all programs, both intervention and control participants continued to receive traditional Medicare coverage (US federal government supported fee for service payments), with an additional fixed negotiated fee per participant per month paid to the programs for each intervention participant. The impact of the various programs tested in the MCCD on medical expenditures, quality of care, and health service utilization has previously been reported [27]–[29].

The program described in the current study was designed and implemented by Health Quality Partners (HQP), (http://www.hqp.org), a not-for-profit health care quality research and development organization, and one of the programs participating in the MCCD. Some findings from the current study were previously included in a report to the US Congress, in which a 25% reduction in all-cause mortality among intervention participants compared to usual care participants was observed for the HQP program [29]. The current study was undertaken to more thoroughly evaluate the program's effect on mortality up to 5 y following enrollment.

## Methods

The study protocol (Text S1) and CONSORT checklist (Text S2) are provided as supporting information. Though nested within the larger MCCD, this study's design and execution were undertaken by the authors independently of CMS or MPR. The national and HQP program-specific evaluation plans designed by MPR for the MCCD have previously been reported [30],[31].

### Participants

All participants randomized into the HQP program from the start of the MCCD in April 2002 through March 2008 are included in this study. Traditional, fee for service Medicare beneficiaries with Parts A (hospitals, skilled nursing facility, hospice, home health care) and B (physician services, outpatient care, home health services) insurance coverage, residing in eastern Pennsylvania, 65 y of age and older, with heart failure, coronary heart disease, asthma, diabetes, hypertension, or hyperlipidemia, and receiving care at a primary care practice agreeing to work with the HQP program, were eligible to participate in this study. No minimum prior health care utilization or hospitalization was required for eligibility. Exclusion criteria included dementia, end-stage renal disease, schizophrenia, active cancer (except skin) in the prior 5 y, life expectancy less than 6 mo, and current or imminent residence in a long-term care facility. Individuals at very low risk for future health complications based on a pre-enrollment assessment were also excluded from the study. In September 2006, a protocol change made a pre-enrollment assessment of low risk an additional exclusion criterion, because interim evaluations indicated that control group participants in this stratum were not utilizing enough health care services to allow for a sufficient realization of savings in the intervention group to offset program costs.

In the US, Medicare is provided in two basic forms: (1) fee for service coverage (traditional Medicare), funded and administered by the federal government, and (2) managed care coverage (Medicare Advantage), funded by the federal government, but sold and administered by private health plans. In areas of the country where insurers offer Medicare Advantage plans, Medicare beneficiaries can choose between these two types of coverage. Medicare Advantage plan members often have financial incentives to use providers within networks recognized by the health plan, and such plans may provide various forms of care coordination or chronic disease management services. By contrast, traditional Medicare beneficiaries can choose to receive their care from any participating Medicare provider and can switch providers at any time without financial penalties. Traditional Medicare, to date, lacks significant care coordination or chronic disease management benefits. All the participants in the current study were beneficiaries receiving traditional US Medicare.

Potential study subjects were referred to the study from participating primary care practices. Practices were assisted to utilize administrative billing data to identify Medicare beneficiaries that might be eligible for the study based on ICD9 diagnosis codes, age, and insurance information. Primary care providers reviewed the patient list generated from billing data queries and selected patients to refer to the study. Outreach to potentially eligible patients was undertaken by HQP by way of a mailed letter of introduction and follow-up phone calls inviting referred patients to learn more about the study.

### Developing a Network of Participating Primary Care Practices

A network of primary care practices was developed by meeting with and describing the HQP program and the MCCD to hospitals, physician-hospital organizations, independent physicians associations, and individual practices. The basic requirements of practices agreeing to participate include: (1) responding to communications about their patients initiated by the nurse care managers on an as needed basis, (2) making the office medical records available to the nurse care managers and chart auditors, and (3) assisting in case-finding potentially eligible individuals on their patient panels, using billing system reports or extracts, or other mutually agreed to processes. The program was designed and promoted as easy to use and free of burdens related to: paperwork, recurring authorizations or pre-certifications, routine case reviews, or administrative tasks.

Practices were encouraged to “test drive” the program by initially referring a small number or select set of patients meeting eligibility criteria. Offices were not required to sign a contract or commit to a minimum length of participation and there were no financial transactions involved. It was explained to offices that by virtue of the randomization process roughly half of their referred and randomized patients would be assigned to the control (usual care) group and half to the intervention group; underscoring that half of all patients from their practice that participated would not receive any extra services. Business Associate agreements committing HQP to safeguard the privacy and confidentiality of the personal health information provided by the practices were executed.

During the time period of this study, 93 primary care practices in and around the 4,662-km^2^, four-county service area of eastern Pennsylvania (Bucks, Montgomery, Lehigh, and Northampton) agreed to participate. Patients of these practices received most of their acute care services from seven hospitals owned by six different health systems. Most practices solicited (greater than 80%) agreed to participate except for those affiliated with two hospital-owned, multi-practice networks (one operating as a Preferred Provider Organization) that declined to participate, citing their desire to: (1) implement and manage their own care coordination programs to enhance their ability to negotiate with health plans, and (2) maintain more direct control over such programs.

Participating practices varied widely in terms of size (most had four or fewer primary care providers), use of electronic records, and organizational affiliation (most were independent). In the past few years, an increasing number of practices have implemented some form of the patient-centered medical home (PCMH); designed to support primary care physicians to improve the proactive coordination and tracking of patient care, typically involving the use of information systems, disease registries, and care team models. There have been no observed barriers, operational difficulties, or decreased interest in collaborating with the HQP program as the result of offices adopting the PCMH.

### Ethics

CMS administered the overall conduct of the MCCD. As previously reported, “The Secretary of Health and Human Services, acting through the CMS, determined that the overall demonstration and evaluation met all criteria in both the Common Rule and National Institutes of Health's Exemption Number 5 for exemption from institutional review board review for research and demonstration projects on public benefit and service programs.” [27] (page 604). All participants provided written informed consent prior to study enrollment. HQP separately sought and received approval of the Institutional Review Board of Doylestown Hospital (Doylestown, Pennsylvania, US) for the present study.

### Classification Prior to Randomization

After providing consent, but prior to study randomization, each participant was classified using two different schema: primary diagnosis and risk stratum. The nurse care management supervisor made the determination of the primary diagnosis. For participants with only one of the chronic health conditions required for study eligibility, that condition was considered the primary diagnosis. For participants having more than one qualifying diagnosis, the condition judged most likely to precipitate a future hospitalization, on the basis of the participant's clinical measures, self-management skills, disease-specific symptoms, and hospital utilization in the prior 6 mo, was chosen as the primary diagnosis.

Eligible participants were also classified into discrete categorical risk strata [31] (page 13). The first step in the algorithm HQP used to determine risk strata, is an assessment of geriatric-related risks using the Sutter Health Questionnaire (used with permission, Cheryl Phillips) [32],[33]. A number of domains are covered in this questionnaire including: self-rated health, number of medications taken, change in weight, falls, health care utilization in prior 6 mo, living arrangement, care giver status, activities of daily living, instrumental activities of daily living, ancillary health care services used, physical activity level/mobility, chronic illnesses, depression, and tobacco use. Individuals scoring at or above a level 3 on the Sutter instrument were defined as the high-risk stratum for this study. Individuals scoring below this breakpoint on the Sutter tool received a second, disease-specific risk assessment developed by HQP, which was used to classify participants into one of three additional risk strata: moderate, low, and very low. Individuals in the very low risk stratum were excluded from study participation from the outset, and those in the low-risk stratum were also excluded beginning in September 2006.

In the course of administering the pre-randomization Sutter Health Questionnaire a numeric risk score (total score) was also calculated. This score was used to augment the outcomes analysis in this study by creating risk subgroups according to total score tertiles: lower, middle, and upper, defined by total scores of <15, 15–35, and >35, respectively.

### Intervention

Participants randomized into the control group received the usual care afforded to traditional Medicare beneficiaries and following notification of their study group assignment, had no further contact with HQP. Participants randomized into the intervention group were provided the HQP model of community-based nurse care management. This model was previously described in a report by the MCCD contracted evaluators [31]. The HQP program was developed over several years in multiple care delivery settings and incorporated a broad portfolio of evidence-based preventive and care management interventions delivered longitudinally by nurse care managers in collaboration with local health care and social service providers. A detailed listing of the elements of this intervention is provided as a supplemental table (Text S3). Nurse care managers used a database developed by HQP to track their activities and participant contacts as well as key assessments and clinical data on participants. Additional paper-based documentation and assessment tools were organized and maintained in participant chart records. All intervention group participants received additional assessments to identify their physical, functional, cognitive, psychological, behavioral, social, and environmental needs. Participants determined to be in the high-risk stratum, on pre-randomization assessment, received a comprehensive, in-home geriatric assessment involving 15 specified elements, including: physical assessment (HQP), Index of Independence in Activities of Daily Living (Katz), Mini-Mental State Exam (Folstein), Clock Drawing Test (Heinik et al.), Geriatric Depression Screen-Short Form (Sheikh and Yesavage), Nutritional Risk Assessment – Nutrition Screening Initiative (NSI), violence screening (HQP), alcohol abuse screening using the CAGE Questionnaire (Ewing), behavioral and caregiver assessment, home environment safety checklist, Numeric Pain Scale (Jacox), sleep, incontinence, assessment of immunizations and preventive screenings, and psychosocial support needs (HQP).

Regardless of enrollment risk strata assignment, however, the nurse care manager developed an individualized plan for each participant. Three factors were used to establish priorities for this plan: (1) the participant's self-articulated primary concerns and unmet needs, (2) findings from risk assessments and evaluations (initial and repeated), and (3) the participant's motivational readiness. Though a structured instrument was not used to assess an individual's motivational readiness, care managers were trained to recognize stages of readiness for change and to apply interventions appropriate to each stage using the Transtheoretical Model of Behavior Change (Prochaska and DiClemente).

Interventions typically incorporated into an individualized plan included: education, symptom monitoring, medication reconciliation and counseling for adherence, and help identifying, arranging, and monitoring community health and social service referrals. Group interventions such as curriculum-based education; structured lifestyle and behavior change programs for weight loss; weight loss maintenance; exercise classes for improving strength and increasing physical activity; and a balance and mobility program for fall prevention were also provided directly to participants by the nurse care managers. Nurses collaborated with the participants' primary care physicians and specialists on an as needed basis to help participants achieve target clinical goals and receive appropriate and timely preventive care according to guidelines. Collaboration also allowed early identification of new or worsening conditions or symptoms, and facilitation of timely medical interventions in an effort to prevent disease exacerbation, hospital admissions, and unnecessary use of the emergency department.

The nurse care managers were community based and, depending on the size of a practice's patient panel, served patients from multiple primary care practices. Participant encounters consisted of in-person visits, group sessions, and telephone contacts. In-person encounters occurred in the participants' homes, physicians' offices, and other accessible community settings, such as HQP's offices, hospitals, community centers, libraries, and faith-based organizations. Contact frequency was determined by participant need with a minimum standard of a monthly contact. On average, participants received 17.4 total contacts per year during the period included in the current study. More than half of all contacts were made in-person either as one-to-one meetings or as group classes. Individualized intervention plans were continuously updated to match the dynamic needs of participants and their caregivers. Once enrolled into the program, intervention participants received services until they died, moved out of the area, requested disenrollment, had a change in insurance coverage making them ineligible for the demonstration, or were placed in a care environment in which the nurse care manager felt they were unable to significantly add to the effectiveness of care (e.g., hospice placement). Once fully trained, each care manager served 85 to 110 participants depending on caseload complexity, geographic distribution, experience, and phase of study recruitment.

In 2007, a protocol of intensified follow-up was added for participants transitioning home or to another level of care upon discharge from hospital. The protocol established guidelines by which nurses provided timely coordination and communication with hospital and post-hospital care providers. The goals were to ensure well informed, safe, and expeditious discharge plans, perform timely patient follow-through on discharge instructions, reconcile medications, and identify and address any errors, omissions, or contraindications in order to prevent readmissions and other serious adverse events.

Program implementation and reliability were supported by careful nurse selection and recruitment practices, pre-service training, ongoing coaching and supervision, structured protocols, explicit operating procedures, clearly articulated performance standards, and a system of data management and statistical process control analysis and reporting to support organizational decision making. A further description of the management elements of this model is provided as a supplemental table (Text S4). This set of management practices has been described as “core implementation components” [34]. Program improvement efforts were ongoing and continuous and resulted in numerous refinements to the program over the course of its implementation within the MCCD.

### Objectives

The main objective of this study was to determine whether HQP's model of community-based care management, as implemented in the MCCD, is associated with a reduction in all-cause mortality overall and within subgroups of risk strata and primary diagnoses. Another objective was to determine whether there was an intervention-associated reduction in all-cause mortality within subgroups defined by tertiles of a numeric risk score obtained on intake assessment using the Sutter Health Questionnaire. The main reasons to explore treatment effect within these subgroups included: (1) refine future program eligibility criteria to direct resources to those that benefit most from the intervention, and (2) permit comparison of impacts on health outcomes to financial outcomes using similar or identical subgroups used by MPR and CMS in their separate and independent financial analyses. It was hypothesized that participants classified as belonging to one or more high-risk subgroups were more likely to demonstrate an intervention-associated reduction in mortality over the follow-up period of this study. The pre-specified and post hoc analyses of the study are summarized in Table 1.

**Table 1:** Outcomes and subgroup analyses specified in the study protocol.

Characteristic	HQP Study Protocol	Pre-specified or Post Hoc
Overall Mortality (all participants)	Primary outcome	Pre-specified
Risk stratification level	Subgroup analyses	Pre-specified
Risk score	Subgroup analyses	Decision to analyze by tertile subgroups was post hoc
Primary enrollment diagnosis	Subgroup analyses	Pre-specified
Clinical cardiovascular risk factors	Secondary outcomes	Not part of current study because data collection is still underway

### Outcomes: Pre-specified

The primary outcome of this study was the risk of death from any cause among intervention participants compared to control participants overall and within subgroups defined by risk strata and primary diagnosis. Vital status as of March 31, 2009 was assessed for all participants. The data source used to establish death was the online Social Security Death Master File (SSDMF) (http://www.ssdmf.com). Social security numbers obtained from participants following informed consent and prior to randomization were used to check vital status in the SSDMF.

### Outcomes: Specified Post Hoc

Analyzing deaths within subgroups defined by tertiles based on the numeric risk score obtained from the Sutter Health Questionnaire was not pre-specified in the study plan. After the study began, but before analysis commenced, this outcome was added. On the basis of random samples, we estimated an overall error rate of 3%–5% in the assignment of participant risk stratum. This rate was due to mistaken Sutter level determinations resulting from the manual tallying of risk scores and variation in the optional use of “flags” (specific question responses defined in the Sutter Questionnaire), which can, if four or more are present, result in increasing the Sutter level by one level. The numeric risk score of the Sutter Health Questionnaire when calculated retrospectively by computer using questionnaire data fields was more reliable.

The risk score derived from the Sutter Questionnaire is obtained in the first step of a multi-step process required for final risk stratum assignment. The risk score is a numeric variable (range in our data: 1–136, mean 29). The use of a computer calculated risk score alone, if predictive of outcomes, could offer a more streamlined, reliable, and efficient method of risk classification, potentially improving future program operations.

### Sample Size

The original minimum enrollment recommendation for MCCD study sites (686 in total; 343 participants each for treatment and control groups) made by MPR as part of their sample size estimation was based on the expected impact of effective interventions on hospitalization as described in MPR's study plan for the MCCD [30]. These original sample size calculations were not based on estimated impacts on mortality. Given the actual number of overall participants in this study (1,736), the observed probability of death in the control group (0.129), and the observed unadjusted hazard ratio (0.75), with alpha set at 0.05, this study is estimated to have a power of 58% for analysis of overall mortality risk using the Cox proportional hazard method. Similarly calculated power estimates for subgroups were lower, with the exception of the upper risk tertile (power = 67%) and coronary heart disease (power = 77%) subgroups.

### Randomization and Blinding

The study was conducted as a parallel group, randomized, controlled trial. Randomization took place at the individual participant level within each of the risk strata determined by HQP prior to enrollment (high, moderate, and low) using a secure website managed by MPR. Participants were randomized on a 1∶1 (intervention: control) basis. All randomization was done offsite by MPR per a protocol established by them and approved by CMS using randomly generated, concealed 4-digit “strings” of treatment-control assignments. By excluding strings of all treatment or all control assignments runs of more than six consecutive assignments to any group were prevented. The random assignment result was available to the program site via the website almost immediately. For practical reasons, study group assignment was not blinded.

### Statistical Methods

All participants randomized into the trial from its start in April 2002 through March 2008 are included in the outcome analysis according to their original study group assignment. The primary outcome (vital status) on all randomized participants (regardless of early program discontinuation) was collected and analyzed through March 31, 2009. The observation period available for each individual participant ran from his or her date of randomization through March 31, 2009 or the completion of a full 5 y of observation (whichever occurred first). Discontinuation from study participation occurring before observation endpoints were reached, for any reason including lost to follow-up, was not a reason for exclusion from the outcome analysis.

Mortality over time was plotted using the Kaplan-Meier method with *p*-values calculated using the log-rank test. The Cox proportional hazard method was used to calculate hazard ratios. Covariates selected for inclusion in Cox regression models had a significant association with the risk of death in univariate analysis and a recognized association with mortality (sex, age group, primary diagnosis, perceived health rating, number of medications taken, hospital stays in the past 6 mo) or failed to reach significance in univariate analysis, but are widely acknowledged to have a strong association with death (tobacco use). The proportional hazard assumptions for Cox regression models were tested using Schoenfeld residuals and no violations were identified (all *p*-values ≥0.05). Subgroup analyses include significance testing of interaction effects using likelihood ratio testing to compare proportional hazards models with a subgroup-treatment interaction term to one without.

Comparison of categorical data was performed using Fisher exact test. Comparison of continuous data was performed using the Student's *t* test when data was normally distributed or Wilcoxon's rank sum method when data significantly departed from a normal distribution. All values for *p* were calculated using two-sided tests. Statistical tests were performed using Stata/MP 10.1 for Macintosh (http://www.stata.com).

## Results

### Participant Recruitment and Flow

**Figure 1:**
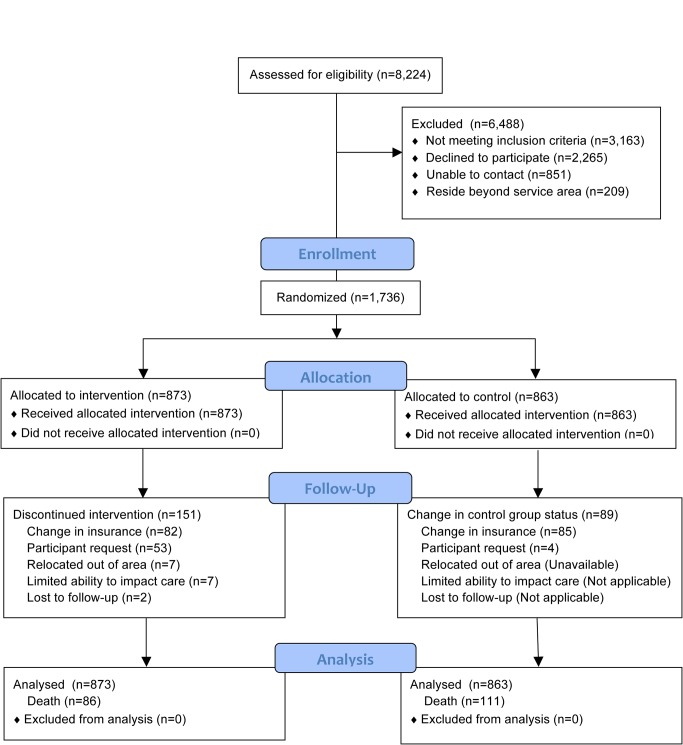
CONSORT flow diagram.

Of all patients referred (*n* = 9,362), sufficient data to attempt assessing study eligibility were available on 88% (*n* = 8,224). The CONSORT flow of participants through allocation, follow-up, and analysis is represented in Figure 1. Overall, 43% (1,736) of individuals confirmed to be eligible and living within the program service area agreed to participate in this study. Of the 2,265 (57%) eligible participants declining to participate, reasons for refusal were captured on 2,134 (94%). These are summarized in Table 2. The number of participants randomized into the study by year is presented in Table 3.

**Table 2:** Reasons eligible individuals declined study participation, *n* = 2,265.

Reason	Number	Percent
Satisfied with current care	1,054	46.5
Too busy	307	13.6
Too old	230	10.2
Overwhelmed with present number of providers	195	8.6
General mistrust of solicitations	177	7.8
Initially accepted then changed their mind	171	7.5
Reason not captured	131	5.8

**Table 3:** Study enrollment by year.

Group	Year Enrolled							
	2002	2003	2004	2005	2006	2007	2008^a^	Total
Control	134	273	231	128	23	74	0	863
Intervention	136	276	236	128	24	73	0	873
Total	270	549	467	256	47	147	0	1,736

a Through March 2008.

Of participants randomized to the intervention group, 151 (17%) prematurely ceased receiving the intervention before reaching an observation endpoint or experiencing an outcome event (death). The most frequent reason for early withdrawal from the intervention was a change in health care insurance coverage (*n* = 82). Eighty-five participants in the control group also had a change in health care insurance coverage. In most cases, these changes resulted from individuals opting to enroll in a private health insurance administered Medicare plan (Medicare Advantage). The median time from enrollment to program discontinuation for any reason for intervention and control participants was 557 and 560 d, respectively. All of these participants are included in the outcome analyses.

The mean follow-up for both control and intervention groups was 4.2 y. In this study, 815 (47%) participants reached the 5-y observation endpoint (alive) and 731 (42%) participants reached the March 31, 2009 endpoint (alive). Altogether, 197 (11%) died prior to reaching these endpoints.

### Baseline Data

Baseline characteristics for all participants and those belonging to the subgroups of high-risk stratum, upper risk tertile, and primary diagnosis of coronary heart disease are presented in Table 4. Overall, among the study population the mean age was 75 y, 61% were female, 31% lived alone, 17% rated their health as fair or poor, 14% said they were depressed in the prior 3 mo, and 22% reported a fall in the prior year. Participants in the high-risk stratum subgroup had an average age of 78 y, 73% were female, 67% lived alone, and 40% rated their health as fair or poor, 35% were depressed in the prior 3 mo, and 40% had fallen in the prior year. By contrast, participants in the coronary heart disease subgroup were less likely to be women (39%) and less likely to live alone (27%). Baseline characteristics of intervention and control participants are shown in Table 5

**Table 4:** Baseline characteristics of participants overall and selected subgroups.

Characteristic	Classification	*n* (%) All	n (%) High-risk Stratum	Upper Risk Tertile *n* (%)	*n* (%) Coronary Heart Disease
**Participants ** ***n***		1,736	505	568	300
**Sex - Female**		1,057 (61)	370 (73)	416 (73)	117 (39)
**Age in years** (mean ± SD)		74.8±6.5	78.2±7.1	78.1±6.9	76.5±6.7
**Age group - years**	65–69	502 (29)	85 (17)	87 (15)	61 (20)
	70–74	433 (25)	78 (15)	94 (17)	66 (22)
	75–79	418 (24)	130 (26)	156 (27)	72 (24)
	80–84	256 (15)	124 (25)	141 (25)	72 (24)
	85+	127 (7)	88 (17)	90 (16)	29 (10)
**Perceived health**	Excellent	304 (18)	33 (7)	43 (8)	42 (14)
	Good	1,124 (65)	273 (54)	327 (58)	198 (66)
	Fair	266 (15)	161 (32)	160 (28)	56 (19)
	Poor	42 (2)	38 (8)	38 (7)	4 (1)
**Living alone**		546 (31)	336 (67)	401 (71)	82 (27)
**Depressed in prior 3 mo**		244 (14)	171 (34)	176 (31)	49 (16)
**Fall in prior year**		374 (22)	200 (40)	210 (37)	61 (20)
**Limited mobility**		162 (9)	153 (30)	155 (27)	34 (11)
**Unintended 4.54-kg+weight loss**		72 (4)	41 (8)	42 (7)	11 (4)
**ADL score** (mean ± SD)		0.8±2.1	2.2±3.4	2.0±3.3	1.0±2.5
**IADL score** (mean ± SD)		1.1±2.4	3.0±3.5	2.7±3.5	1.4±2.8
**Need help to complete risk survey**		155 (9)	119 (24)	123 (22)	31 (10)
**Tobacco use**		80 (5)	17 (3)	18 (3)	18 (6)
**Chronic conditions** (mean ± SD)		3.8±1.9	5.2±2.2	5.0±2.2	4.5±1.8
**Nursing home stay ever in past**		22 (1)	20 (4)	20 (4)	4 (1)
**Number of medications**	5 or more	971 (56)	383 (76)	421 (74)	217 (72)
	2 to 4	624 (36)	111 (22)	132 (23)	74 (25)
	1	108 (6)	10 (2)	14 (2)	6 (2)
	None	33 (2)	1 (0)	1 (0)	3 (1)
**Physician or clinic visits in past 6 mo**	4 or more	468 (27)	216 (43)	233 (41)	93 (31)
	2 or 3	807 (46)	218 (43)	251 (44)	136 (45)
	1	401 (23)	62 (12)	75 (13)	61 (20)
	None	60 (3)	9 (2)	9 (2)	10 (3)
**ER visits in past 6 mo**	3 or more	19 (1)	13 (3)	14 (2)	4 (1)
	2	54 (3)	39 (8)	42 (7)	15 (5)
	1	240 (14)	116 (23)	126 (22)	47 (16)
	None	1,423 (82)	337 (67)	386 (68)	234 (78)
**Hospital stays in past 6 mo**	4 or more	18 (1)	17 (3)	17 (3)	4 (1)
	2 or 3	49 (3)	30 (6)	35 (6)	12 (4)
	1	177 (10)	98 (19)	101 (18)	38 (13)
	None	1,492 (86)	360 (71)	415 (73)	246 (82)
**Primary diagnosis**	Heart failure	98 (6)	58 (11)	60 (11)	—
	Coronary heart disease	300 (17)	110 (22)	116 (20)	300 (100)
	Diabetes mellitus	316 (18)	119 (24)	122 (21)	—
	Hypertension	673 (39)	150 (30)	187 (33)	—
	Asthma	81 (5)	36 (7)	39 (7)	—
	Hyperlipidemia	268 (15)	32 (6)	44 (8)	—
**Risk stratum**	High	505 (29)	505 (100)	490 (86)	110 (37)
	Moderate	1047 (60)	—	76 (13)	170 (57)
	Low	184 (11)	—	2 (0)	20 (7)
**Risk tertile**	Upper	568 (33)	490 (97)	568 (100)	116 (39)
	Middle	600 (35)	15 (3)	—	111 (37)
	Lower	368 (33)	0 (0)	—	73 (24)

ADL, activities of daily living; ER, emergency room; IADL, instrumental activities of daily living; SD, standard deviation.

**Table 5:** Baseline characteristics of participants by study group.

Characteristic	Classification	*n* (%) Intervention	*n* (%) Control
**Participants**		873	863
**Sex - female**		537 (62)	520 (60)
**Age in years** (mean ± SD)		74.7±6.5	74.9±6.5
**Age group - years**	65–69	260 (30)	242 (28)
	70–74	212 (24)	221 (26)
	75–79	207 (24)	211 (24)
	80–84	129 (15)	127 (15)
	85+	65 (7)	62 (7)
**Perceived health**	Excellent	151 (17)	153 (18)
	Good	566 (65)	558 (65)
	Fair	136 (16)	130 (15)
	Poor	20 (2)	22 (3)
**Living alone**		276 (32)	270 (31)
**Depressed in prior 3 mo**		125 (14)	119 (14)
**Fall in prior year**		181 (21)	193 (22)
**Limited mobility**		82 (9)	80 (9)
**Unintended 4.54-kg+weight loss**		38 (4)	34 (4)
**ADL score** (mean ± SD)		0.8±2.2	0.8±2.1
**IADL score** (mean ± SD)		1.1±2.4	1.1±2.4
**Need help to complete risk survey**		80 (9)	75 (9)
**Tobacco use**		37 (4)	43 (5)
**Chronic conditions** (mean ± SD)		3.8±1.9	3.8±2.0
**Nursing home stay ever in past**		12 (1)	10 (1)
**Number of medications**	5 or more	512 (59)	459 (53)
	2 to 4	301 (34)	323 (37)
	1	44 (5)	64 (7)
	None	16 (2)	17 (2)
**Physician or clinic visits in past 6 mo**	4 or more	232 (27)	236 (27)
	2 or 3	402 (46)	405 (47)
	1	206 (24)	195 (23)
	None	33 (4)	27 (3)
**ER visits in past 6 mo**	3 or more	10 (1)	9 (1)
	2	27 (3)	27 (3)
	1	109 (12)	131 (15)
	None	727 (83)	696 (81)
**Hospital stays in past 6 mo**	4 or more	7 (1)	11 (1)
	2 or 3	25 (3)	24 (3)
	1	90 (10)	87 (10)
	None	751 (86)	741 (86)
**Primary diagnosis**	Heart failure	50 (6)	48 (6)
	Coronary heart disease	138 (16)	162 (19)
	Diabetes mellitus	176 (20)	140 (16)
	Hypertension	348 (40)	325 (38)
	Asthma	39 (4)	42 (5)
	Hyperlipidemia	122 (14)	146 (17)
**Risk stratum**	High	252 (29)	253 (29)
	Moderate	528 (60)	519 (60)
	Low	93 (11)	91 (11)
**Risk tertile**	Upper	289 (33)	279 (32)
	Middle	302 (35)	298 (35)
	Lower	282 (32)	286 (33)

ADL, activities of daily living; ER, emergency room; IADL, instrumental activities of daily living.

### Mortality Analyses

Overall, 86 (9.9%) intervention participants and 111 (12.9%) control participants died during the study period, representing a 25% lower relative risk of death (unadjusted hazard ratio [HR] 0.75 [95% CI 0.57–1.00], *p* = 0.047) among the intervention group. When covariates for sex, age group, primary diagnosis, perceived health, number of medications taken, hospital stays in the past 6 mo, and tobacco use were included in the model, the adjusted HR was 0.73 (95% CI, 0.55–0.98), *p* = 0.033.

**Figure 2:**
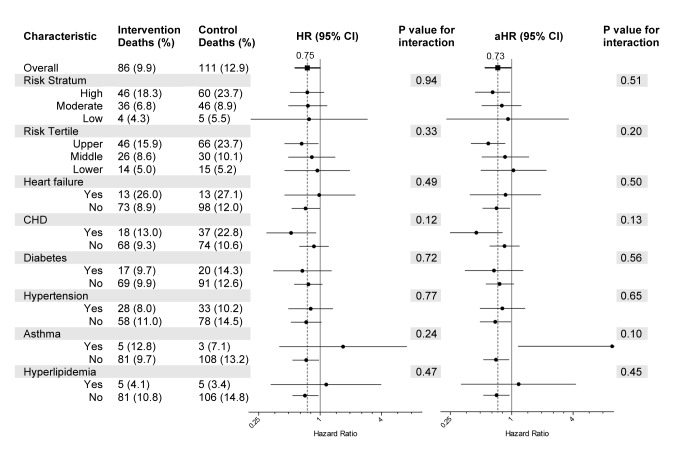
Subgroup analyses. Deaths and tests for interaction by subgroup. HRs and adjusted HRs (aHR) along with 95% CIs are represented by forest plots with *x*-axis in log 2 scale. The regression model used for the aHR includes covariates for sex, age group, primary diagnosis, perceived health rating, number of medications taken, hospital stays in the past 6 mo, and tobacco use. CHD, coronary heart disease.

**Figure 3:**
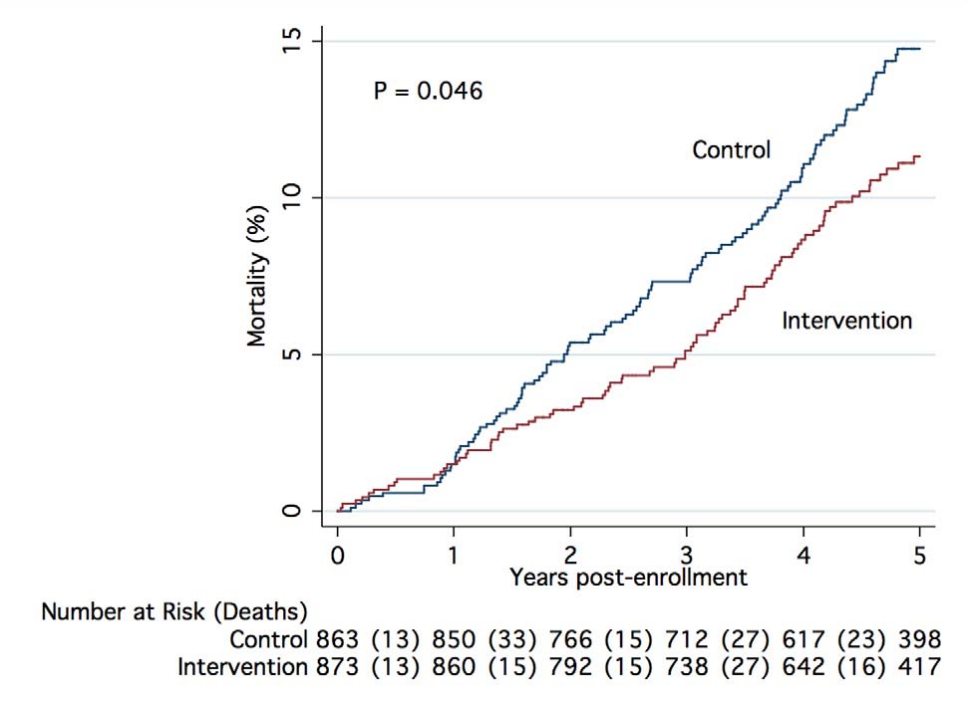
Kaplan-Meier estimate of cumulative mortality up to 5 y from enrollment. The plot includes results for all participants randomized into the study, (unadjusted data), with *p*-value calculated using the log-rank test.

The number and percentages of deaths, graphical representation of the unadjusted and adjusted hazard ratios, and the *p*-values from tests of subgroup-treatment interaction overall, and for all subgroups are provided in Figure 2. Subgroup analyses did not demonstrate statistically significant interaction effects for any subgroup. A Kaplan-Meier plot and log rank test comparing intervention and control groups overall is shown in Figure 3.

There was a 100% match between deaths known to nurse care managers in the intervention group and deaths identified in the Social Security Death Master File (SSDMF).

### Adverse Events

No known program-related adverse events were identified.

## Discussion

### Main Findings

This study provides evidence that the model of community-based nurse care management tested is associated with a reduction in all-cause mortality among chronically ill older adults participating in fee for service Medicare in the US. The strengths of the study include: a randomized controlled trial design (with randomization at the individual participant level), model implementation in collaboration with a broad array of primary care providers across a sizeable geographic region, a long follow-up period, and use of the intention-to-treat method of analysis.

In the setting of small sample sizes and low statistical power, the subgroup analyses are best viewed as exploratory. There is a suggestion that participants in the upper risk tertile and those with a diagnosis of coronary heart disease may experience a greater survival benefit from the program. The lack of statistically significant subgroup-treatment interaction, however, indicates the need for caution when interpreting apparent differences between subgroups. The study helped confirm the feasibility of collecting self-reported information for intake risk assessment to identify subgroups that may be more likely to benefit from this intervention.

The finding of an increased hazard ratio for intervention participants in the asthma subgroup was unexpected and corresponds to a total subgroup size of 81 (intervention and controls) with only three control and five intervention deaths. Retrospective reviews of HQP chart records for the five intervention participants who died revealed: one died of multiple myeloma while receiving hospice care, one of unknown causes during sleep, one of complications apparently arising from a hospital misadventure resulting in acute renal failure and sepsis, one of severe chronic obstructive lung disease while receiving hospice care, and one died more than a year after discontinuing participation in the HQP program following a change in health insurance coverage. Given the small subgroup size and event counts, Kaplan-Meier plot pattern (not shown), log rank test *p*-value of 0.472, and the findings noted on chart review, we believe there is little evidence of a program-related association or mechanism for an increased risk of death among intervention participants within the asthma subgroup.

### Limitations

CMS did not make any claims data available to HQP for program operations, performance improvement, or research purposes and did not permit HQP to have any contact with control participants following randomization. HQP's MCCD-related funding consisted of a per participant per month fee for care coordination services with no additional support for research activities associated with the demonstration. Given these limitations, the authors could not directly analyze differences in medical expenditures or health care service utilization between treatment and control groups, though these analyses have been previously reported by others [28],[29].

Evaluating the impact of care coordination models on mortality was not the primary objective of the MCCD, and the sample size for this study overall and for most subgroups was smaller than optimal for this purpose. A small sample size increases the risk of failing to identify a true difference in survival between treatment and control groups when one actually exists (a type II error), but small subgroups also increase statistical volatility such that small numbers of events or small differences in regression covariates can have an exaggerated effect on results. A likely case in point was the unexpected finding of an increased HR among intervention participants in the asthma subgroup.

The study focused on one unique model of community-based care management in a single geographic region of the US. Participants in this study were predominantly white and only a small proportion was believed to be economically poor (though socioeconomic, racial, and ethnic data were not collected in this study). Testing the generalizability of this model among more racially, ethnically, culturally, and economically diverse populations and in other geographic regions is an important research imperative. Until such research is undertaken, it will be impossible to know to what extent the demographic profile of participants in the current study was a determinant of the effectiveness of the model.

### Interpretation

The current study provides the strongest evidence to date that a model of community-based nurse care management can reduce the mortality rate for chronically ill older adults. The study also supports the broader concept that, at least under some circumstances, nurses playing a more intensive role in the longitudinal care of chronically ill older adults can improve the long-term health outcome of this population. This point had not been well established in previous research. A few studies from Europe, of smaller size and shorter duration than the study reported here, are associated with a survival advantage among older adults receiving various types of home visits by nurses [35]–[38]. To date, most studies of care coordination models in the US, including those applying the chronic care model, focusing on primary care redesign, medical homes, or transitions of care, have either not reported mortality as a separate outcome or have demonstrated no impact on mortality [39]–[53]. Two studies from the US that did not randomize individual participants and had other methodological limitations have reported improved survival for recipients of nurse care management provided within primary care settings [54],[55].

In reports by the CMS-contracted evaluators of the MCCD, the impact of the HQP model of community-based care management on health expenditures and health service utilization varied by pre-randomization risk, defined either by the risk stratification method used in the current study or by a combination of diagnoses and health service utilization. With all enrollees included (low, moderate, and high risk), no statistical difference in medical expenditures or health service utilization between the intervention and usual care groups has been observed [28],[29]. When analysis is restricted to the subgroup of the high risk stratum (as defined in the current study) intervention participants were reported to have 29% fewer hospitalizations and 20% lower expenditures than individuals assigned to usual care ([27], pages 614–615). Among a subgroup of participants with heart failure, coronary heart disease or chronic obstructive pulmonary disease, and at least one hospitalization in the prior year, the intervention group had 39% fewer hospitalizations, 37% fewer emergency room visits, a 36% decrease in total Part A and Part B Medicare expenditures and a net savings to Medicare (after HQP program fees) of US$397 per participant per month [29]. As a result of these findings, starting October 2010, with continued CMS support, eligibility for this study was changed and HQP began prospectively enrolling higher-risk beneficiaries—individuals with a history of heart failure, coronary heart disease, chronic obstructive pulmonary disease, or diabetes, and at least one hospitalization (for any reason) in the year prior to study randomization.

Favorable impacts on health service utilization and expenditures among higher risk participants and reduced overall mortality suggest that this model of community-based nurse care management works by reducing avoidable complications that increase both the use of acute health care services and the risk of death. This may have been accomplished, in large part, by supporting participants to better adhere to physician-initiated treatment plans concordant with evidence-based guidelines. Nurse care managers also prompted primary care providers whenever “clinical inertia” [56] or deviations in treatment plans prevented participants from achieving guideline defined goals.

Apart from being an important health outcome in its own right, improved survival, when driven, as in this case, by a preventive intervention, is very likely accompanied by other important improvements in health, functional status, and quality of life, though these have not been measured over the long-term in the MCCD. To assess the full value of this model, future research should include longitudinal measures of self-rated health, functional status, and quality of life. As one example, the HQP program includes interventions related to advance directives education and advanced care planning, but to date, there has been no published analysis comparing intervention and usual care participants with respect to expenditures for skilled nursing facility, hospice, or end-of-life care.

The HQP model of community-based care management has been tested on a regional scale in a health care delivery environment typical of much of the US for over 9 y and found to be compatible with and complementary to the work that primary care practices are increasingly engaged in to develop a patient-centered medical home. The current study also provides evidence that it is feasible to implement this program in collaboration with small, independent primary care practices. Office practices with five or fewer physicians accounted for about 73% of primary care practices in the US in 2003–2004 (with 46% of practices consisting of only one or two physicians) [57]. Smaller practices have been reported to be less likely to use patient-centered medical home processes indicating that effective chronic disease management interventions through office-based efforts alone may be especially challenging for such practices [58].

Forty-three percent of eligible individuals contacted agreed to participate in this study and 57% declined. Future experimental research must recognize and adequately accommodate the cost and time required to case-find and enroll sufficient numbers of participants into studies of this kind. Non-experimental evaluations of replication or scalability efforts, not requiring randomization or informed consent, would likely see greater rates of enrollment among those eligible. Analysis of the variation in enrollment rates between sites could potentially offer insights into how best to optimize engagement and enrollment of eligible individuals. Use of aggregated health care data, if available, would greatly improve the efficiency and effectiveness of case-finding and participant recruitment.

It is not known whether the model used in the current study would be effective in other countries having different demographic profiles, socioeconomic conditions, health care insurance, or health care delivery systems. The model's attributes of being community-based, requiring relatively modest start-up capital, and its use of nurses, may make it an approach worth testing in some global health settings. The program as implemented in this study utilized collaboration with primary care physicians therefore locations with reduced availability or access to primary care services could see diminished effectiveness. In some areas, shortages of nurses with sufficient training or experience may preclude implementation of the program. Whether other types of health workers can be trained to substitute for nurses in this model is unclear, but the current study used highly experienced nurses as care managers and the use of alternative providers may not yield similar results.

In light of the many care coordination and disease management models that have failed to demonstrate comparable improvements in health outcomes, it seems likely that a degree of fidelity to model design and implementation will be necessary for reproducible effectiveness. Efforts to maintain such program fidelity may conflict with the need for local adaptations to allow implementation in a new environment. The authors believe that core elements contributing to this program's effectiveness include: (1) delivering a broad set of services that match the preventive health needs of the targeted population, (2) frequent longitudinal in-person contacts with participants, (3) collaboration with primary care providers, and (4) training, management, and performance monitoring capabilities.

The program of community-based care management tested in the current study appears to be a valuable addition to the primary care of appropriately selected chronically ill older adults. Efforts to more broadly test the adaptability, scalability, and generalizability of this model seem warranted. Future progress in this area will, like many innovations in biomedical science and public health, probably require multiple, well-designed, longitudinal trials.

## Supporting Information

Text S1 **Study protocol.**
(PDF)

Text S2 **CONSORT checklist.**
(DOC)

Text S3 **Elements of the intervention.**
(DOC)

Text S4 **Elements of program management.**
(DOC)

## Abbreviations

CMS: Centers for Medicare and Medicaid Services
HQP: Health Quality Partners
HR: hazard ratio
MCCD: Medicare Coordinated Care Demonstration
MPR: Mathematica Policy Research, Inc
