# Supplementary material for: Effect of a Community-Based Nursing Intervention on Mortality in Chronically Ill Older Adults: A Randomized Controlled Trial
Source: PLoS Med. 2012 Jul 17;9(7):e1001265. doi: 10.1371/journal.pmed.1001265 (PMC3398966; doi:10.1371/journal.pmed.1001265)
Supplement: Text S1 — Study protocol. (PDF) [file pmed.1001265.s001.pdf]

Research Protocol

Population – The currently proposed study will *initially* evaluate the outcomes of Medicare Coordinated Care Demonstration participants randomized at the HQP site during its first 6 years of operations; 1,736 chronically ill older adults. The intervention group (n = 873) was offered an integrated set of geriatric care coordination, disease management, and preventive services provided by community-based nurse care managers working collaboratively with primary care providers and other community resources. The control group (n = 863) received usual care without the services of the HQP community-based nurse care program.

Subsequent analyses to increase the length of follow-up time and to include more recently enrolled participants to the study population would be undertaken on a rolling annual basis for up to 5 years from this study's IRB approval.

Data Sources – Vital status will be assessed for all demonstration participants. The principal data sources for this information will include the Social Security online working file, (access to which has been provided to HQP by CMS to confirm initial and continued Medicare eligibility among demonstration participants), and the publicly available online Social Security Death Master File (SSDMF). The completeness of information available from these data sources will be checked by comparing deaths identified from these data to deaths known to have occurred among intervention group participants whose histories are well known to HQP's nurse care managers. If any discrepancies are identified between these sources of information, additional review of existing clinical information and/or confirmation with the Bureau of Health Statistics and Research of the Pennsylvania Department of Health (to which an application to access protected data will be made following IRB approval) will be sought.

Clinical data from primary care and other health providers was collected in the routine course of providing nurse care management services to intervention group participants including blood pressure, body weight, and laboratory values related to total cholesterol, LDL cholesterol, HDL cholesterol, triglycerides, HgbA1c, and fasting glucose through primary care medical record abstraction, electronic data sources, and hard copy reports provided by patients. When appropriate, clinical data were also obtained by direct measurement of blood pressure or weight by the nurse care manager.

Medical chart abstraction of clinical measures for control group participants by HQP staff was planned for, approved (by CMS), and initiated at the start of the demonstration, but had to be discontinued in 2007 due to lack of funding. Nonetheless, where sufficient data exists, it may be possible to use this information in the current study to compare trends in clinical cardiovascular risk factors between control and intervention groups.

Data Management and Security – Existing clinical and vital status data are kept on computers secured by password protected logins and maintained in encrypted folders (directories). Control group clinical data resides on only two computers; the PI's laptop and a firewall protected server located at HQP's main office at 875 North Easton Road, Suite 10, Doylestown, PA 18902. The office requires visitor sign-in and staff escort upon entry and has an automatic alarm system to detect and deter forced entry during off hours.

Intervention group clinical data are regularly accessed and used by HQP nurse care managers in their daily work with patients. Replicated copies of these data exist on laptops used by each nurse and synchronized with data on a central server. Other authorized HQP staff including the

subinvestigator, chief of information technologies, and senior data analyst also have access to these data.

Updated vital status data as it is collected is stored in the HQP central server in the main office and by virtue of database replication\synchronization is available on the laptops of all staff.

Privacy and Confidentiality – Participants consented at the time of their initial enrollment into the MCCD, to allow HQP access to and appropriate utilization and sharing of personal health information from any source of medical or behavioral health information.

All reports, papers, and presentations deriving from this study will only provide aggregated results for the populations analyzed and will not reveal any individual participant identification or information that could be traced to or associated with any individual participant.

Analysis –

Study Outcomes:

The primary outcome of this study is all-cause mortality analyzed on an intention to treat basis. This analysis will be done comparing control and intervention groups overall and by subgroups based on geriatric risk score, risk stratification level, and principal enrollment diagnosis.

A secondary outcome of this study is change in clinical cardiovascular risk factors among study participants comparing pre-enrollment to subsequent (e.g., last follow-up) values for body weight, blood pressure, total cholesterol, LDL cholesterol, HDL cholesterol, triglycerides, and HgbA1c.

Statistical Tests:

Mortality analysis will be conducted on an intention to treat basis and no participant completing initial randomization through March 2008 will be excluded from this analysis or attributed to any category other than their original assignment to the intervention or control group. The Cox proportional hazard method will be used to calculate hazard ratios. Covariates included in the regression model will be significantly associated with the risk of death in univariate analysis and/or be used to adjust for difference in the baseline distribution of key attributes between control and intervention groups. Comparison of categorical data (e.g., baseline geriatric risk factors) will be performed using Fisher's exact test. Comparison of continuous data (e.g., systolic blood pressure) will be performed using the student's t-test when data is normally distributed or Wilcoxon's rank sum method when data significantly departs from a normal distribution. All values for p will be calculated using two-sided tests. Statistical tests will be performed using Stata/MP 10.1 for Macintosh (Stata Corporation).
