# Supplementary material for: Effect of a Community-Based Nursing Intervention on Mortality in Chronically Ill Older Adults: A Randomized Controlled Trial
Source: PLoS Med. 2012 Jul 17;9(7):e1001265. doi: 10.1371/journal.pmed.1001265 (PMC3398966; doi:10.1371/journal.pmed.1001265)
Supplement: Text S3 — Elements of the intervention. (DOC) [file pmed.1001265.s003.doc]

Interventions (including Assessments, and Screenings):

*The following interventions are provided by HQP Nurse Care Managers with participants through in-person contacts (home visits and office visits and in groups) and through telephone monitoring and follow-up.*

| **Intervention** | **Description** | **Application** | **Protocol / Standard** |
| --- | --- | --- | --- |
| Intake Assessment | Sutter Health Questionnaire (SHQ) - a validated geriatric risk assessment; patient self-report, nurse administered; scored by algorithm and identifies patients at high risk for death, hospitalization, nursing home placement or other adverse events | All participants | Completed following patient consent and prior to randomization; nurse administered based on patient self-report; nurse reviewed for omissions, discrepancies, conflicts |
| Initial Geriatric Assessment | Comprehensive, multidimensional in-home assessment of physical, functional, cognitive, psychological, behavioral, social and environmental needs. Specific tools used to conduct this assessment are described in Methods : Intervention section | All intervention participants who scored ‘high risk’ on the SHQ | Completed within 30 days of randomization utilizing the structured screening and assessment tools |
| Individualized Plan | Developed initially and updated each encounter based on: the patient’s self-identified primary concerns and unmet needs; findings from their initial and ongoing assessments; and the patient’s motivational stage of change | All intervention participants | Developed following initial geriatric assessment and during each structured encounter |
| Action Plans | Individualized plan that identifies when the patient is to call the nurse care manager, the physician, and when to call 911 (general and disease specific) | All intervention participants receive a general action plan and condition specific plan(s) as appropriate | Initially within 30 days of randomization and updated and reviewed with the patient on each subsequent encounter |
| Ongoing Assessments and Screenings | Ongoing assessments and screenings utilizing structured tools for the standard encounter and screening for depression, domestic violence, abuse, neglect and preventive care and immunizations. | All intervention participants | Structured assessments completed monthly utilizing the HQP structured encounter; annual screenings and preventive care according to guidelines |
| Medication Reconciliation and Management | The process of identifying and creating an accurate list of the patient’s current medications; reconciling errors/omissions with the prescribing physicians; assessment of patient adherence (obtaining and taking medications as prescribed), and assisting in organizing, managing and educating the patient about their medication regimen to support adherence; identify root causes for non-adherence and utilize collaborative problem solving to address barriers | All intervention participants | Medication review and reconciliation on the initial assessment and during each subsequent contact and during care transitions |
| Care Transitions | Intensification of assessment, coordination and visits by the nurse care manager when the patient is admitted/discharged from hospital, nursing home and home care; timely assessments and visits with patients to ensure safe and well coordinated care transitions with follow through on instructions, medications, and treatment plans | Intervention participants with a visit to an emergency department or admission to a hospital | Protocol guides coordination with healthcare providers, follow up calls and frequency of visit with patient during the care transition periods |
| Education and Self-Management Training | Comprehensive structured curriculum for disease specific education and self-management training for asthma, cardiovascular diseases, and diabetes – provided one to one or in a small group of participants | Condition specific; based on assessment finding of the patient’s knowledge and skills, needs, priorities and risks | Provided for all patients and customized based on disease state, patient needs and priorities with ongoing assessment and tracking through a structured education plan |
| Assessment and counseling for behavior change | The Transtheoretical Model of Behavior Change is used by care managers to continually assess patients’ motivational stage for behavior change (lifestyle behaviors, self-management and self-monitoring skills) and supporting patients with appropriate cognitive or behavioral strategies | Assess participants’ stage of behavior change and match interventions to their stage of readiness | Assess and provided based on the patients’ needs and priorities |
| Nutritional Education and Counseling | Individualized patient education and counseling for low sodium; reduced fat; carbohydrate counting; meal planning, portion control, calories. | Patient and condition specific based on motivational stage and individual need | Assess and provided based on the patients’ needs and priorities |
| Physical Activity Education and Counseling | Individual patient education and counseling to adopt a more active lifestyle as well as more formal exercise prescriptions | Patient and condition specific based on motivational stage and individual need | Assess and provided based on the patients’ needs and priorities |
| Stress Management Education and Counseling | Assess the factors that are contributing to stress and identify the resources and techniques to manage stress | Patient specific | Assess and provided based on the patients’ needs and priorities |
| Quit smoking Education and Counseling | Assess readiness to quit; provide appropriate cognitive or behavior strategies and collaborating with primary care physicians for pharmacological treatment | Participants who smoke | For people who currently smoke, assess readiness to quit at each encounter |
| Advance Directives Education | Identify the presence of current advance directives (durable power of attorney for health care decisions, and living will) and provide patients education regarding their right to self-determination and preferences for choosing a decision maker and to designate their individual preferences for care at the end of life. | All intervention participants | Identify presence and location of patients’ advance directives initially and periodically re-assess and review advance directives with patients |
| Advanced Care Planning | Anticipation of patients’ future care needs and assisting patients and families with planning to meet those needs – treatment, end of life options, living situation, etc. | All intervention participants | Consider advance care planning based on patient age and nature of illnesses and patient specific situation |
| Medical Management with Physicians | Collaboration with physicians to report new or worsening symptoms, abnormal findings, psychosocial issues and recommendations regarding treatment plan and/or routine preventive care | All intervention participants as needs are identified | Care Manager contacts physician by telephone, fax or physician preferred method of contact |
| Psychosocial Needs Assessment & Information and Referral | Assess patients’ needs for services, Federal state and county services (pharmaceutical assistance, in home care), non-covered services (DME, meals, private care), service monitoring and follow up, behavioral health services | All intervention participants as needs are identified | Initial and ongoing as needed |
| Coordinating Care | Based on patients’ needs collaboration with family, and other health and social service providers to communicate changes in treatment plan, medication management, home environment and safety, monitoring of services and providers involved in the patients care | All intervention participants as needs are identified | Initial and ongoing as needed |
| LEARN® Weight Management Group | A 16 week, structured group program facilitated by care managers, addresses the multiple factors associated with sustainable weight loss | Intervention participants with a BMI > 30 in the ‘action’ stage of change | Periodic assessment of patients’ motivational stage of readiness for weight loss through this behavioral intervention |
| Weight Loss Maintenance Group | A monthly group program that is care manager facilitated and provides ongoing education and support for participants who have lost weight and for weight maintenance. Education and reinforcement on behavioral strategies, nutrition, physical activity and regular weight monitoring | Intervention patients who have completed a weight loss program or who want to keep from gaining weight | Recommend as a follow on to the LEARN Weight Management Program |
| Seated Exercise Group | Weekly group program that is supervised by a care manager and guided by video of seated exercises and stretching as a way for participants to learn and practice daily physical activities | All intervention participants who are functionally able to safely participate | Encourage attendance for participants who are appropriate for participating in seated exercise in a community based group setting |
| Diabetes Conversation Map® | A five week small group interactive workshop, facilitated by care managers for diabetes education, and self-management skill development based on current practice guidelines | Intervention participants with a diagnosis of diabetes | Encourage participation by participants with a diagnosis of diabetes, for support, education, skill development and problem solving related to the multidimensional problem of diabetes |
| FallProof™  Groups | An intensive 10 week 18 session group program facilitated by nurses that includes a pre/post program evaluation for balance and mobility assessment and training | Participants with history of falls | Assess incidence of falls each contact; if positive for falls, consider for FallProof™ program, physical therapy or home exercise program |
