# Supplementary material for: Effect of a Community-Based Nursing Intervention on Mortality in Chronically Ill Older Adults: A Randomized Controlled Trial
Source: PLoS Med. 2012 Jul 17;9(7):e1001265. doi: 10.1371/journal.pmed.1001265 (PMC3398966; doi:10.1371/journal.pmed.1001265)
Supplement: Text S4 — Elements of program management. (DOC) [file pmed.1001265.s004.doc]

Management Elements:

*The following management elements were used to support delivery of the community-based care management program.*

| **Management tool** | **Description** | **Major Elements included** |
| --- | --- | --- |
| Pre-service training | A comprehensive and closely managed six – nine month orientation and training program that involves didactic education, self-learning, participant observation, role play, case review; while building a full patient caseload. | - Initial and ongoing assessments and screenings – risk screenings nutrition; fall, domestic violence, abuse, neglect, exploitation, mental status, cognition, depression, suicide, substance, home safety, medications - Patient engagement - Person centered approach - Visit preparation - Behavior change theory - Motivational interviewing - Evidence-based clinical practice guidelines - Provider communication - Patient goal setting - Patient education curriculum - Action plans - Information systems - Best practices in time management - Patient and caseload reports - Community resources - Group program interventions – LEARN®, Weight loss maintenance, seated exercise, FallProof™, Diabetes Conversation Map® |
| Coaching and supervision | - Following pre-service training; regular and ongoing individual meetings between the supervisor and care manager for caseload monitoring and review. - Weekly team huddles for communication updates, continuing education and nursing development, case and standards review | - Review of all patients with nurses, utilizing quality reports with special focus on complex patients and those recently hospitalized; - Periodic chart reviews to evaluate interventions and documentation; - Structured observation visits to observe pre-visit preparation, nurse-patient interactions, including person-centeredness; assessment, screening interventions, education, goals setting, etc. - CM consultation with nursing leads for advise and support in managing patients with difficult, complex, and safety issues (medical, psychiatric, social environmental); - Regular performance review and feedback |
| Protocols / Guidelines | - Protocols to guide CM processes and interventions; - Evidence-based clinical practice guidelines | - Policies, procedures, and standard operating procedures for   - patient screenings (e.g. depression, abuse, neglect, exploitation), and for positive findings;   - assessments,   - care transitions,   - medication management and reconciliation;   - timing of follow up contacts;   - guidelines for cardiovascular disease, diabetes, chronic lung disease, preventive care, physical activity, weight loss, smoking cessation |
| Performance standards, metrics and reports | Role specific standards of performance reinforced by guidelines, protocols, operating procedures | Evaluated with approximately 200 metrics using a data system with near real time reports, supervisory observation visits and patient surveys and call backs |
